# Dismantling inequities to end the black maternal mortality crisis in the United States

**DOI:** 10.1186/s12939-025-02488-1

**Published:** 2025-04-28

**Authors:** Ameneh Safarzadeh

**Affiliations:** University of Massachusetts Amherst, Amherst, MA USA

## Abstract

Black maternal mortality in the United States remains alarmingly high—Black women are still more than three times as likely to die from pregnancy-related causes than White women. This crisis is not due to individual choices or access alone, but to deeply rooted structural inequities, including systemic racism, policy exclusion, and the neglect of Black-led care models. While efforts like Medicaid expansion and the Black Maternal Health Momnibus Act aim to address these gaps, they often fall short by relying on narrow, one-size-fits-all solutions. This commentary uses Critical Health Equity theory and the Intersectionality-Based Policy Analysis (IBPA) framework to examine how current policies may unintentionally reinforce the very inequities they seek to eliminate. It argues that achieving real equity in maternal health requires more than reform—it calls for a fundamental shift in who holds power, whose knowledge is valued, and how care is delivered. A new framework is proposed that centers Black leadership, supports community-led research, and promotes culturally safe, justice-oriented care.

## Introduction (Merged with current status & causes)

Black maternal mortality in the United States remains one of the most alarming and persistent indicators of racial injustice. In 2022, the maternal mortality rate for Black women was 49.5 deaths per 100,000 live births—more than 2.5 times higher than the rate for White women (19.0) and nearly four times that of Asian women (13.2) [1]. Although this marks a decrease from the pandemic peak in 2021 (when the national rate reached 32.9), the disparities remain stark and deeply entrenched. Decades of public health awareness and targeted initiatives have not closed this gap. Black birthing people continue to face systemic devaluation within the U.S. healthcare system [2].

The COVID-19 pandemic further magnified these inequities. Black communities faced disproportionately high infection and death rates, compounding risks for pregnant individuals due to delayed care, pre-existing comorbidities, and institutional neglect [3]. Importantly, these disparities are not new. Even prior to the pandemic, Black women—regardless of income or education—were at higher risk. A Black woman with a graduate degree remains more likely to die from pregnancy-related causes than a White woman with only a high school diploma [2].

These outcomes cannot be understood through individual behavior, access, or socioeconomic status alone. They stem from deeply rooted structural inequities—including systemic racism, implicit bias, chronic disinvestment in Black communities, and the persistent exclusion of Black voices in healthcare leadership. Structural determinants such as residential segregation, underfunded healthcare infrastructure, environmental injustice, and discriminatory policy all interact to create dangerous conditions for Black mothers [4].

Geography also plays a critical role. Many Black women live in “maternal care deserts”—regions with few or no obstetric providers—particularly in states that have resisted Medicaid expansion [5]. The fragmentation of postpartum healthcare coverage further compounds these risks, often cutting off essential services during a vulnerable period [6].

As Bowleg (2017) powerfully asserts, referring to these disparities without acknowledging their structural and political roots risks reinforcing the same inequities we aim to dismantle. A critical health equity lens challenges us to consider how knowledge is produced, whose experiences are validated, and which paradigms dominate maternal health research and policy [7, 8].

This commentary therefore moves beyond description to structural critique. It interrogates dominant maternal health narratives that individualize risk or blame provider-level failure, instead offering a justice-based lens grounded in epistemic equity, structural reform, and Black community leadership [7, 8]. Achieving true maternal health equity requires not only improving existing programs but reimagining the systems and frameworks that define care itself.

## Structural critique of maternal health policy frameworks

Efforts to address the Black maternal mortality crisis in the United States have gained national visibility. Yet, most policy interventions continue to operate within frameworks that are limited, incremental, and politically cautious [9]. These initiatives often fall short not because they are poorly implemented, but because they are grounded in technocratic epistemologies that reduce systemic issues to technical challenges—thus sidelining the lived experiences and expertise of Black women and birthing people [10, 11].

Guided by Bowleg’s (2017) critical health equity stance, this section applies the Intersectionality-Based Policy Analysis (IBPA) framework to critically evaluate how even well-intentioned policies can reproduce inequities. Specifically, we examine how the invisibilization of racism, erasure of historical trauma, and failure to interrogate power asymmetries allow structural harms to persist beneath the surface of equity discourse [7, 12, 13].

### Federal legislation: the black maternal health Momnibus act

The Black Maternal Health Momnibus Act—originally introduced in 2021 and reintroduced in 2023—comprises a series of bills designed to improve maternal health outcomes through strategies like data modernization, workforce diversification, and investment in community-based programs [14, 15]. Despite its ambitious agenda, the Act is largely rooted in a biomedical model that frames disparities as solvable through better access, expanded coverage, and improved provider training [6, 14, 15].

While the Act includes promising provisions—such as support for culturally congruent care and funding for Black maternal health organizations—it stops short of fundamentally challenging the structural conditions that drive racialized disparities in maternal health. When viewed through the IBPA lens, several critical blind spots emerge:

- Whose knowledge is privileged? The Act depends heavily on federally guided, institutional approaches and medical systems, often overlooking or underfunding grassroots, Black-led initiatives with demonstrated community impact.
- Which intersecting systems of oppression are addressed? While the Act references social determinants of health, it does not meaningfully confront larger structural forces such as the criminal legal system, housing discrimination, or environmental racism—factors that profoundly shape maternal outcomes for Black communities [16].

Ultimately, the Momnibus Act represents an important step forward symbolically and politically, but lacks the transformative vision necessary to dismantle the political economy that underpins maternal health inequity. Without structural accountability and redistributive reform, it risks reinforcing the very systems it seeks to improve [7, 16].

### Medicaid expansion and postpartum coverage

The expansion of Medicaid postpartum coverage under the American Rescue Plan has been widely applauded as a significant policy advancement, granting states the option to extend postpartum care to 12 months. While this represents progress in increasing formal access to care, the impact has been uneven across the country. As of 2024, several states have yet to adopt the extension, and in those that have, the quality and continuity of care often remain unexamined or inconsistent [17, 18].

Crucially, expanded coverage does not necessarily translate into equity in maternal health outcomes.

- Medicaid expansion improves insurance access, but does not address discriminatory provider practices, implicit bias, or institutional neglect.
- It also fails to confront structural drivers of maternal harm, including racially segregated care landscapes, mistrust rooted in historical mistreatment, and the under-resourcing of safety-net hospitals serving Black communities [17–19].

As Bowleg (2017) argues, policies that neglect to challenge dominant ideologies—such as colorblindness and political neutrality—can unintentionally reproduce the inequities they aim to correct. Without structural reform and accountability mechanisms, Medicaid expansion remains a partial solution to a deeply systemic problem [7].

### Marginalization of Black-led models

Community-based programs—such as Black-led midwifery collectives and reproductive justice organizations—offer care that is grounded in ancestral knowledge, relational trust, and trauma-informed frameworks. These models have demonstrated improved outcomes and patient satisfaction, particularly among Black birthing people. Yet, despite their promise, such programs often remain underfunded, unlicensed, or excluded from federal and institutional funding pipelines [20].

Applying the IBPA framework, two critical questions arise:

- How are Black maternal health leaders and advocates positioned in policy development?
- What are the consequences of continuously under-resourcing culturally responsive care models?

The answer is clear: policies that fail to center Black leadership and lived experience will continue to fall short. Without mechanisms for structural accountability, these innovative models will remain marginalized and peripheral to the dominant healthcare system—unable to scale or sustain impact [21, 22].

### Reframing the narrative

To meaningfully address the Black maternal mortality crisis, we must shift from incremental reform to structural transformation. This requires a reframing of how we define problems—and who gets to define solutions. Specifically, we must move from:

- Fixing providers → to rebuilding institutions.
- Expanding access → to redefining care.
- Inclusion by invitation → to shared governance by design.

This narrative shift demands recognition that current maternal health policy often co-opts the language of equity while sustaining the very hierarchies it claims to dismantle. Rhetoric without accountability merely rebrands injustice. Therefore, policy must be interrogated through a racial and reproductive justice lens—not only for what it proposes, but also for what it omits, excludes, or silences [23, 24].

### Reimagining black maternal health: A structural equity framework

The framework proposed in this commentary is grounded in Critical Health Equity theory [7] and the Intersectionality-Based Policy Analysis (IBPA) framework [12]. It intentionally moves away from deficit-based approaches that emphasize individual behavior or isolated access barriers. Instead, it seeks to directly confront the foundational systems—structural racism, marginalization, and epistemic injustice—that continue to shape health outcomes for Black women in the United States.

As Bowleg (2017) emphasizes, health equity cannot be achieved without critically interrogating how knowledge is produced, who is excluded from that process, and which forms of expertise are legitimized. By placing Black leadership at the center, embracing community-generated evidence, and advancing data collection practices grounded in political consciousness, this framework seeks to dismantle institutional neglect and promote a justice-centered model of maternal care [25, 26].

### Integrating data gaps into structural reform

A critical component of this framework is the integration of data justice. Traditional maternal health data collection often fails to account for the full scope of Black birthing experiences. Specifically, it overlooks:

- Racialized experiences of obstetric racism;
- Intersectional risk factors based on geography, immigration status, disability, or LGBTQ + identity;
- Community knowledge and non-clinical outcomes such as dignity, agency, and cultural safety [13, 24].

To address these omissions, we propose the following measures:

- Mandatory disaggregated data collection by race, region, and socioeconomic status;
- Community-based data governance, ensuring that Black-led organizations have ownership and decision-making power over how data is designed, collected, and applied;
- Federal mandates requiring states to report maternal health outcomes beyond the narrow 42-day postpartum window currently used in many systems.

These reforms are necessary to ensure that data not only inform policy, but reflect the lived realities of the communities most affected.

### Framework pillars

1. Centering Black Leadership and Governance.
  Reallocate decision-making authority to Black maternal health experts, community advocates, and reproductive justice coalitions;
  Move beyond tokenistic inclusion toward structural integration of Black leadership in oversight, funding decisions, and program design.
2. Community-Driven Knowledge and Research.
  Invest in Community-Based Participatory Research (CBPR) led by Black scholars and doulas.
  Validate oral histories, storytelling, and qualitative inquiry as forms of authoritative evidence.
3. Policy Accountability and Reparative Action.
  Require all maternal health policies to undergo Equity Impact Assessments.
  Shift from inclusion to redistribution of resources, ensuring funding prioritizes Black-led, trauma-informed, culturally congruent care.
4. Culturally Safe and Ancestral Care Models.
  Expand access to and investment in Black-led midwifery and doula care programs.
  Legitimize and integrate ancestral knowledge systems and non-Western birthing traditions as fundable, evidence-informed care pathways—honoring cultural continuity and birthing sovereignty.

### Connection to policy recommendations

This structural equity framework serves as a foundation for assessing, critiquing, and transforming current national maternal health policies. It enables a shift from piecemeal reform to systemic change. For example:

- The Momnibus Act, while expansive in scope, must evolve from merely funding discrete projects to facilitating structural redistribution of power and leadership to Black communities.
- Medicaid expansion should be accompanied by enforceable quality benchmarks rooted in anti-racist, equity-driven care—not just broader access.
- Implicit bias training must be embedded within a larger institutional transformation strategy, including accountability measures for racialized harm and structural discrimination.

### Limitations of this framework

While this commentary presents a structural, justice-oriented framework for addressing Black maternal mortality, it is not without limitations. Implementing the proposed reforms will require substantial political will, sustained financial investment, and deep cultural transformation within institutions that have historically resisted equity-driven change.

Moreover, although this framework is intentionally centered on Black maternal experiences, further research is needed to address the distinct and intersecting needs of Black trans and non-binary birthing individuals, immigrants, and people with disabilities. These populations often experience compounded forms of oppression—such as misgendering in clinical settings, immigration-related health barriers, or ableist care policies—that remain underexplored in current maternal health data and discourse.

## Conclusion

This framework repositions maternal health equity not as a technical issue to be solved, but as a political and structural imperative. Addressing Black maternal mortality requires more than improving access or training clinicians—it demands a transformation in how we conceptualize care, distribute power, and validate knowledge.

By integrating principles of data justice, community sovereignty, and structural accountability, this framework offers a path forward for health policies that are not only equity-informed but equity-authored. These policies must not simply aim to include Black women—they must be co-created by them, with leadership, expertise, and lived experience at the center of maternal health governance.

## Data Availability

No datasets were generated or analysed during the current study.

## Abbreviations

ACA: Affordable Care Act
CBPR: Community-Based Participatory Research
CDC: Centers for Disease Control and Prevention
GIS: Geographic Information System
HHS: U.S. Department of Health and Human Services
KFF: Kaiser Family Foundation
SDOH: Social Determinants of Health
U.S.: United States
WHO: World Health Organization
